# A Wide-Angle and Polarization-Insensitive Rectifying Metasurface for 5.8 GHz RF Energy Harvesting

**DOI:** 10.3390/mi16060611

**Published:** 2025-05-23

**Authors:** Zhihui Guo, Juan Yu, Lin Dong

**Affiliations:** 1 School of Information Innovation and Big Data, Shanxi Jinzhong Institute of Technology, Jinzhong 030600, China; 2 Department of Mechanical & Electrical Engineering, Xiamen University, Xiamen 361005, China; 3 Beijing Key Laboratory of Millimeter Wave and Terahertz Technology, School of Integrated Circuits and Electronics, Beijing Institute of Technology, Beijing 100081, China

**Keywords:** rectifying metasurface, energy harvesting, metamaterials absorber, polarization insensitive

## Abstract

This paper presents a rectifying metasurface (RMS) that enables wide-angle, polarization-insensitive wireless energy harvesting in the Wi-Fi frequency range. The RMS consists of a metasurface integrated with rectifying diodes, a low-pass filter (LPF), and a resistive load. In the structural design, the RMS incorporates four Schottky diodes placed on the bottom structure and connected to the top structure through four metallized vias. This configuration facilitates impedance matching between the metasurface and the diodes, omitting the need for conventional rectifier circuits or external matching networks and removing the impact of soldering variations. A 3 × 3 RMS prototype was manufactured and subjected to experimental validation. The measurements confirm that the RMS achieves a peak RF-to-DC conversion efficiency of 68.3% at 5.8 GHz with a 12.5 dBm input power, while maintaining stable performance across a wide range of incident angles and polarization states.

## 1. Introduction

Wireless communication technology has been remarkably developed in recent decades, bringing the Internet of Things into daily life through various sensors. Meanwhile, the proliferation of billions of wireless sensors also presents new challenges to energy supply systems. For portable use, small wireless electronic devices that run on batteries or require wired charging can be very inconvenient [1]. Therefore, exploiting ambient environmental energy has become a viable approach to ensure the long-term operation of wireless sensors. With the growing interest in sustainable power solutions, researchers have explored various ways to harvest energy from the ambient environment, including kinetic, thermal, and solar energy [2,3,4,5,6,7,8]. However, these methods cannot provide continuous electrical supply due to the varying power levels required and the specific harvesting conditions for different types of energy. RF electromagnetic (EM) waves pervade the environment due to wireless communication technology, making it possible to harvest RF energy to supply electric energy for small wireless electronic devices [9,10,11,12,13].

Metasurfaces are artificially engineered structures comprising subwavelength unit cells arranged on a two-dimensional surface. Owing to their ability to locally manipulate electromagnetic wave properties, such as amplitude, phase, and polarization, metasurfaces have found extensive application in electromagnetic shielding and cloaking technologies [14,15,16,17,18,19]. Traditional metasurface absorbers achieve electromagnetic absorption by dissipating the incident energy as heat, typically within the dielectric substrate [20,21,22,23,24,25]. However, such absorbed energy is usually not reused for functional purposes. In recent studies, researchers have explored integrating metasurface absorbers into RF energy harvesting and wireless power transmission systems [26,27,28,29,30,31,32]. Compared with traditional wireless energy harvesting antennas, rectifying metasurfaces (RMSs) possess advantages such as wide-angle coverage, low-profile design, and high efficiency. Previous works have proposed various RMS, which can be mainly divided into three methods: (1) rectifier circuits combined with metasurface arrays using built-in channeling line [33,34,35], (2) rectifier circuits combined with metasurface arrays using matching network [36,37], and (3) rectifier diodes integrated with metasurface units [38,39]. The advantage of the first and second method are that it uses fewer diodes to achieve rectification at low energy densities and reduces the impact of manufacturing accuracy on performance. However, they both require additional rectifier circuit design and extra physical space for placing the rectifier circuit, which not only complicates the structural layout, but also introduces non-negligible energy losses during transmission. The third method avoids the need for rectifier circuit design, extra physical space, and a matching network. However, it typically incorporates diodes mounted directly on the surface structure, which makes the performance highly sensitive to manufacturing accuracy. Even minor deviations in diode positioning can lead to significant degradation in rectification efficiency and shifts in frequency response. The limitations associated with the three existing methods undermine the metasurface’s energy conversion capability and restrict its feasibility for compact integration and large-scale applicability in practical deployment scenarios.

To overcome these limitations, we propose a 5.8 GHz rectifying metasurface designed for wide-angle and polarization-insensitive wireless energy harvesting. The proposed RMS consists of a metasurface integrated with rectifying diodes, a low-pass filter (LPF), and a resistive load. To eliminate the impact of diode soldering on the surface structure, four Schottky diodes are placed on the bottom structure and connected to the top structure through four metalized vias. This approach also avoids the need for rectifier circuit design and a matching network. Meanwhile, energy harvesting efficiency is improved by achieving impedance matching between free space, the metasurface, and the rectifying diodes. Additionally, a 3 × 3 prototype array was fabricated and experimentally characterized to evaluate its RF energy harvesting capability. Experimental results indicate that the RMS maintains over 50% RF-to-DC conversion efficiency from 5.5 to 6.0 GHz, reaching a maximum of 68.3% at 5.8 GHz under an input power of 12.5 dBm. In addition, the proposed RMS demonstrates stable energy collection across diverse incident angles and polarization states, highlighting its applicability in wireless energy capture and ambient power utilization scenarios.

## 2. Rectifying Metasurface Design and Analysis

**Figure 1:**
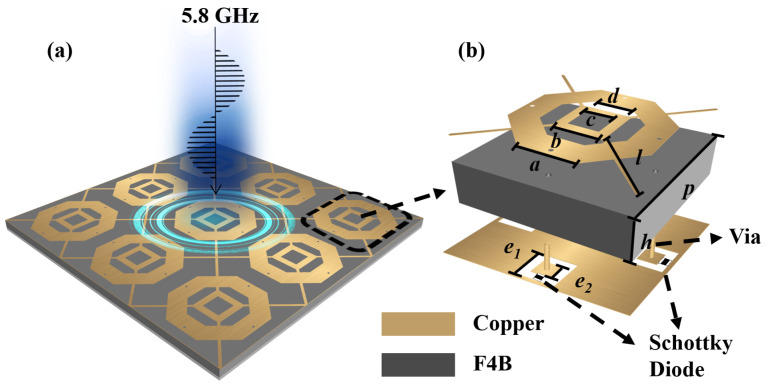
(**a**) Schematic overview of the proposed RMS. (**b**) 3D geometric model of the RMS unit cell. The corresponding structural dimensions are given as: a=6.74 mm, b=6.00 mm, c=3.60 mm, d=4.13 mm, l=9.90 mm, p=20.00 mm, h=3.00 mm, $e_{1}=4.20$ mm, and $e_{2}=2.20$ mm.

The schematic of the proposed RMS for wireless energy harvesting is illustrated in Figure 1a. The unit cell of RMS consists of three layers, where the top and bottom layers are made of 0.035 mm thick copper, as illustrated in Figure 1b. The top structure consists of hexagonal and square-shaped rings interconnected by four DC bias lines, enabling the absorption of arbitrarily polarized EM waves. To prevent interference from the diode on the top structure, four Schottky diodes are installed on the bottom structure. These are connected to the top structure through four metalized vias to rectify the absorbed electromagnetic energy. Additionally, the bottom structure can be used as the ground for the RMS. A 3-mm-thick F4B material ($\varepsilon_{r}=2.65$, tanδ=0.0015) is used as the dielectric layer.

**Figure 2:**
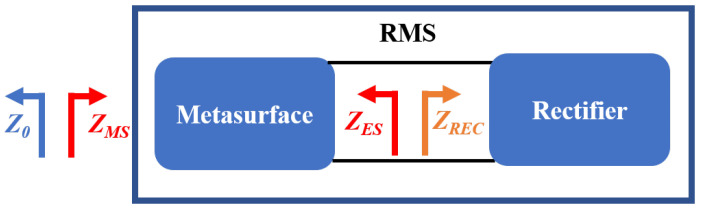
System framework of the proposed RMS and the corresponding input impedance of each module.

Figure 2 illustrates the system framework of the proposed RMS, which integrates a metasurface and rectifying diodes. The RF-to-DC efficiency of RMS is composed of the harvesting efficiency of metasurface ($\eta_{unit}$) and the rectification efficiency of Schottky diodes ($\eta_{AC-DC}$). The harvesting efficiency can be formulated as follows:

(1) $$\eta_{unit}=\frac{P_{H}}{P_{\mathrm{rad}}},$$

where $P_{rad}$ denotes the incident EM energy collected from the ambit environment and $P_{H}$ represents the energy absorbed by the RMS periodic structure and transmitted to the Schottky diodes. The rectification efficiency of Schottky diodes can be formulated as follows:

(2) $$\eta_{AC-DC}=\frac{P_{AC}}{P_{DC}},$$

where $P_{AC}$ is AC energy rectified by the Schottky diodes and $P_{DC}$ represents the out power of RMS. The RF-to-DC efficiency can be expressed as follows:

(3) $$\eta_{RF-to-DC}=\eta_{unit}\cdot \eta_{AC-DC},$$

Achieving high RF-to-DC conversion efficiency requires effective impedance matching between the metasurface ($Z_{MS}$) and free space ($Z_{0}$) to maximize $\eta_{\mathrm{unit}}$. Additionally, the impedance ($Z_{ES}$) of RMS must be conjugate matched with the impedance of the Schottky diodes ($Z_{REC}$) to ensure high $\eta_{AC-DC}$. The $Z_{ES}$ is related to $Z_{REC}$, which is primarily affected by frequency, input power, and load resistance. Therefore, selecting the appropriate input power and load resistance is crucial to achieving the optimal operating statement for the Schottky diode within the RMS.

**Figure 3:**
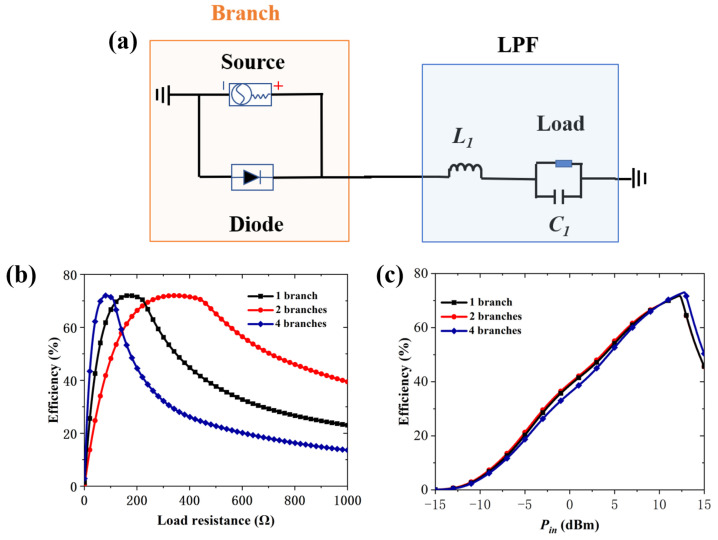
The equivalent circuit model and simulation results of the class rectifier. (**a**) The equivalent circuit of the Class-F rectifier. The rectification efficiency simulation of different parallel branches versus (**b**) load resistance and (**c**) input power.

Figure 3a presents the equivalent circuit of the Class-F rectifier, which consists of a branch and a LPF. The branch comprises a source and a rectifying diode. Compared with other rectifiers, the Class-F topology offers higher conversion efficiency with lower input power requirements [13], making it suitable for enhancing performance and enabling low-power operation in the RMS unit. The Schottky diode HSMS-2860 is selected as the rectifying component. In engineering practice, multiple parallel branches are employed to enhance rectification efficiency and system stability. To evaluate the impact of parallelization, different multi-branch configurations are simulated and analyzed in Advanced Design System (ADS). Figure 3b illustrates how the load resistance influences the rectification behavior of different parallel branches at an input power of 10 dBm. As more parallel branches are introduced, rectification efficiency improves slightly, while the load resistance decreases significantly. A maximum rectification efficiency of 71.9% is obtained by the four-branch configuration with a load resistance of 100 Ω. The lower load impedance facilitates impedance matching, making it suitable for low input power rectification. The four-branch parallel configuration is selected as the equivalent circuit model for the RMS unit, as it balances rectification efficiency and structural symmetry. Meanwhile, the resistance provided by the four diodes enables the metasurface impedance ($Z_{MS}$) to efficiently match that of free space, thereby achieving high absorption. The impact of incident power on the rectification efficiency under optimal load conditions is shown in Figure 3c. According to the simulation, the maximum rectification efficiency reaches 73% at an input power of 12.8 dBm and a load resistance of 100 Ω. Finally, a rectifier consisting of four parallel Class-F branches is adopted in the RMS unit design, with a 100 Ω resistor selected as the load.

**Figure 4:**
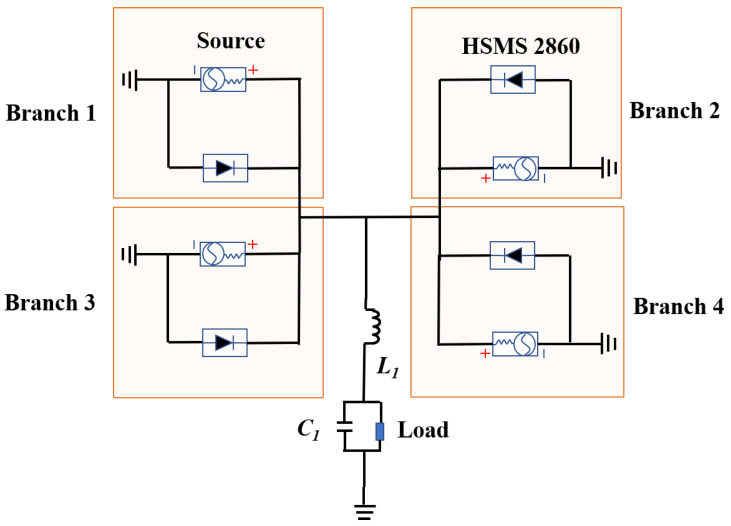
The equivalent circuit model of the proposed RMS unit.

**Figure 5:**
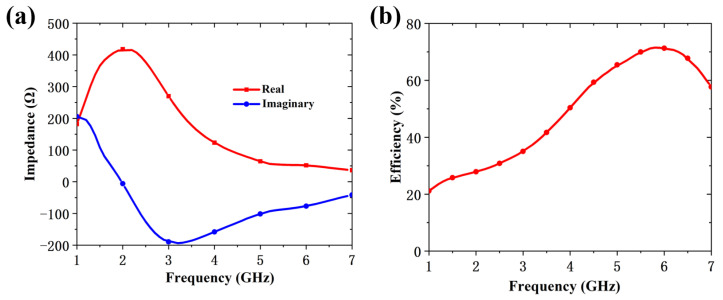
The simulation of the equivalent circuit model. (**a**) Simulated impedance versus frequency. (**b**) Simulated rectification efficiency versus frequency.

Figure 4 illustrates the equivalent circuit of the RMS unit, which consists of four Class-F rectifiers, a low-pass filter, and a load. Following the high-impedance rectenna design method, the $Z_{REC}$ of the RMS does not need to match the standard 50 Ω source impedance. The RMS unit can achieve maximum efficiency by optimizing the input impedance $R_{s}$ + $jX_{s}$. The simulated frequency responses of impedance and rectification efficiency for the equivalent circuit model under 12.8 dBm input and 100 Ω load are plotted in Figure 5. Figure 5a shows the real part of impedance gradually decreases, while the imaginary gradually increasing as the frequency rises from 1 to 7 GHz. The results show that the impedance is 56 − *j*81 Ω at 5.8 GHz. An impedance of 56 − *j*81 Ω is adopted as the input matching condition to achieve optimal rectification at 5.8 GHz. Figure 5b demonstrates that the rectification efficiency surpasses 70% from 5.5 to 6.3 GHz. Additionally, the low-pass filter is used to suppress harmonic waves, which consists of a 47 nH inductance and a 100 nF capacitor.

**Figure 6:**
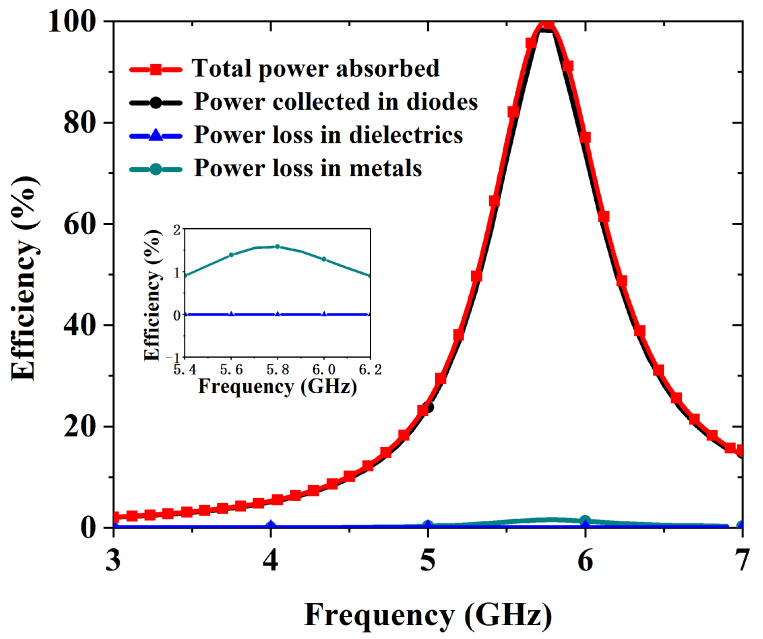
Simulation results of the incident power absorbed by the RMS and the corresponding distribution of power loss.

The impedance $Z_{Es}$ of RMS is set to 52.6 + *j*81 in RMS unit simulation to achieve conjugate matching with $Z_{REC}$. CST Microwave Studio 2020 is used to simulate the RMS unit. The operating frequency can be tuned by modifying the thickness of the dielectric layer and the parameters of hexagonal and square-shaped rings. The resistance introduced by the diodes is transmitted to the top structure through vias, and by adjusting the surface structure, impedance matching between the metasurface and free space can be achieved. Consequently, the placement of the diodes and the spacing between the hexagonal and square-shaped rings directly influence the absorption performance of the RMS. Finally, the structural parameters were iteratively optimized using the built-in PSO (Particle Swarm Optimization) algorithm in CST, allowing the metasurface to achieve high absorption at the target frequency of 5.8 GHz. The optimized design parameters of the RMS unit are detailed in Figure 1. Figure 6 illustrates the distribution of incident power absorption by the RMS, along with the corresponding power losses in the metallic layers, dielectric, and rectifying diodes. The results indicate that the RMS absorbs 99.8% of the incident power at 5.8 GHz, with the absorption remaining above 90% over the frequency range of 5.6 to 5.9 GHz. Notably, the harvesting efficiency ($\eta_{unit}$) reaches 98.2% at 5.8 GHz and, similar to the absorption performance, remains above 90% within the 5.6–5.9 GHz range. The remaining power is dissipated within the dielectric.

**Figure 7:**
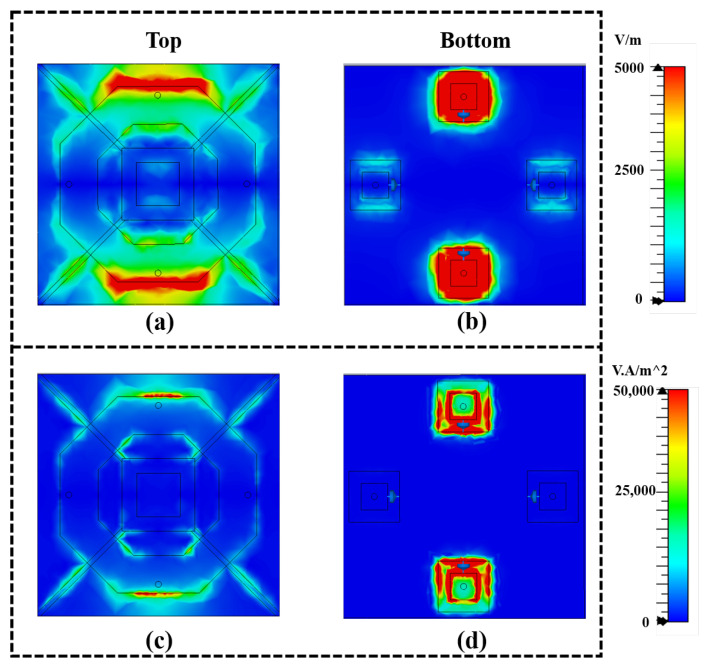
Simulations of (**a**) top view and (**b**) bottom view of the electric field of the RMS, and (**c**) top view and (**d**) bottom view of the power loss distribution of the RMS at 5.8 GHz.

To further analyze the absorption power flow and the operating mechanisms of the RMS unit, the electric field and power flow distributions at 5.8 GHz are simulated under y-polarized wave incidence along the z axis, as illustrated in Figure 7. Figure 7a shows a significantly high electric field near the upper and lower vias of the upper surface structure of the RMS. Moreover, the high electric field resonates on the upper and lower diodes of the bottom structure through the via transmission, as illustrated in Figure 7b. The strong resonance means that the diodes respond most strongly to incident waves, enhancing energy coupling to achieve highly effective absorption at 5.8 GHz. As illustrated in Figure 7c,d, the power flow at 5.8 GHz is primarily concentrated around the bottom diodes, indicating that the incident energy is efficiently directed toward the rectifying components.

**Figure 8:**
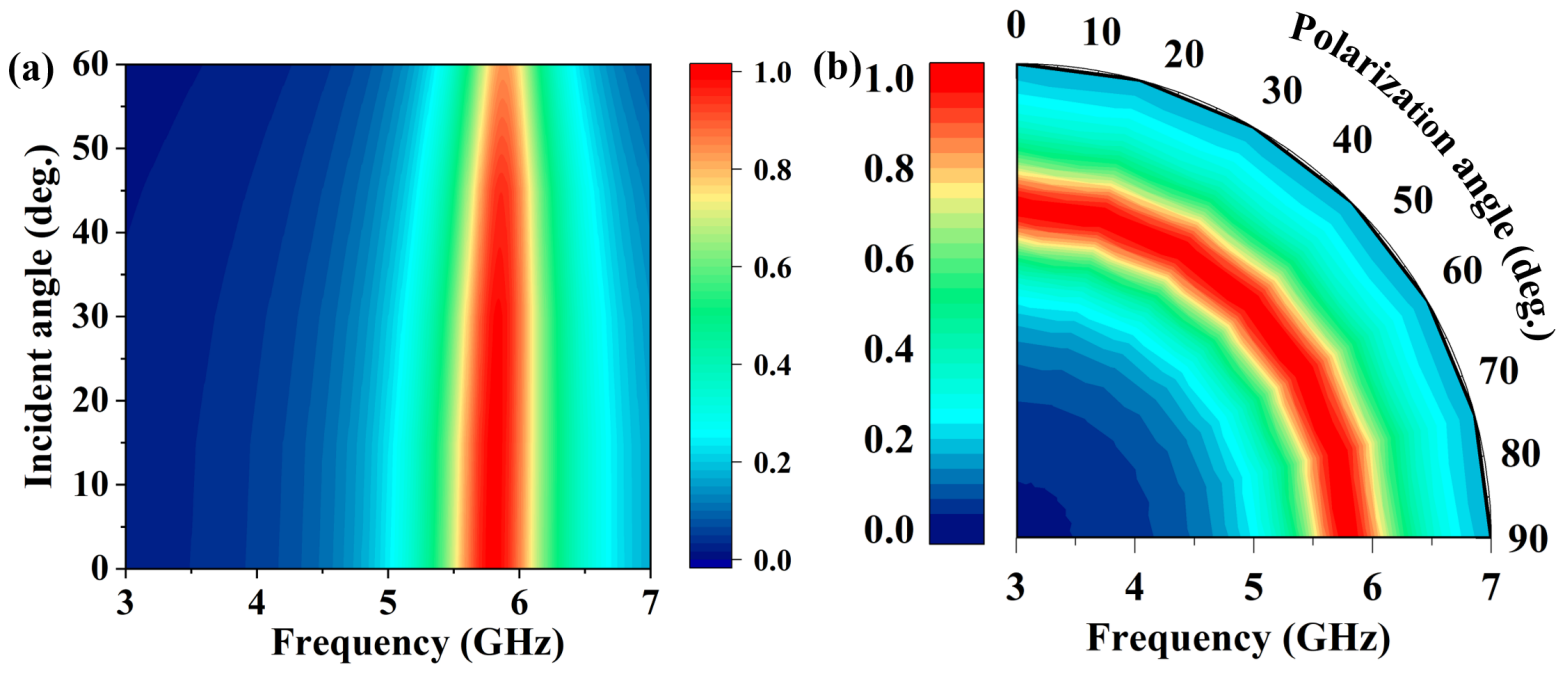
Simulated absorption performance of the proposed RMS. (**a**) Absorption under varying incident angles. (**b**) Absorption under varying polarization angles.

Figure 8 shows the simulated absorption under varying incident and polarization angles. As illustrated in Figure 8a, the RMS demonstrates over 90% absorption at 5.8 GHz for incident angles ranging from 0° to 60°. In addition, Figure 8b presents the absorption under varying polarization directions, where the performance exceeds 98% for angles between 0° and 90°. These results confirm that the RMS achieves stable absorption performance across various incidence angles and polarization states.

## 3. Rectifying Metasurface Measurements and Discussion

**Figure 9:**
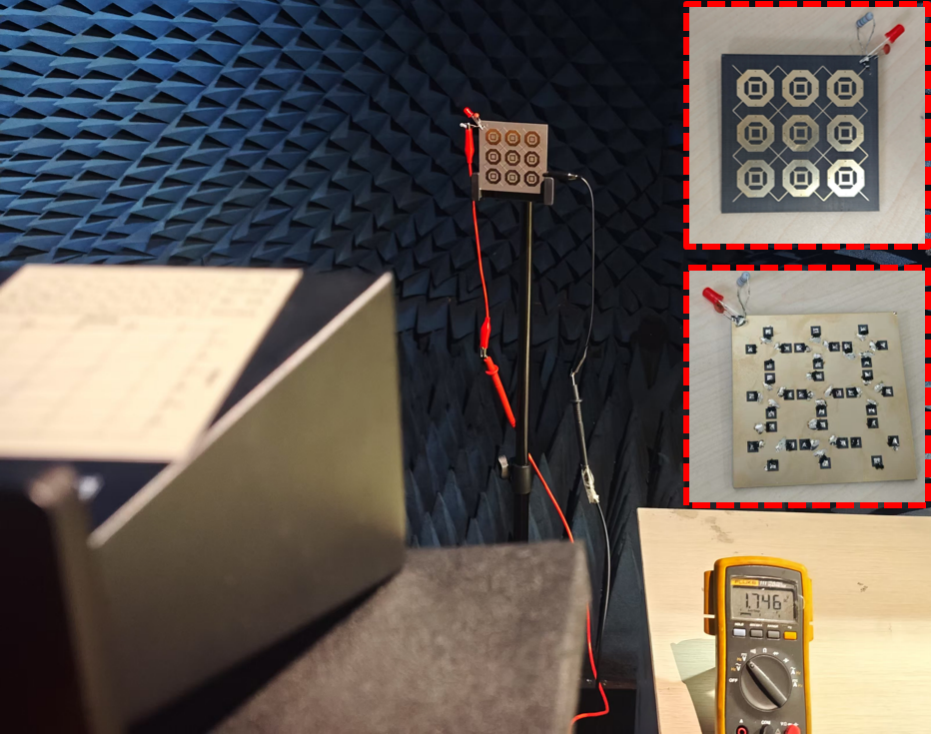
Photograph of the DC measurement setup for the RMS array prototype in an anechoic chamber.

To experimentally validate the proposed RMS design, a 3 × 3 array with overall dimensions of 70.0 × 70.0 mm was manufactured and tested, as illustrated in Figure 9. The RMS was manufactured by printed circuit board (PCB) technology by etching a 0.035 mm copper layer onto an F4B substrate ($\varepsilon_{r}=2.65$, tanδ=0.0015) with a thickness of 3 mm. The diodes were installed on the bottom structure of metasurface through a standard soldering process. Figure 9 also illustrates the measurement process of the RMS in the experimental environment. The measurement setup consists of three parts. First, an RF signal is produced using a signal generator and amplified by a power amplifier. Second, a standard horn antenna, positioned 1 m in front of the RMS, transmits the amplified signal. Third, a digital multimeter is used to measure the DC output voltage across the load. The incident RF power density on the RMS is calculated as:

(4) $$P_{in}=\frac{G_{t}\cdot G_{a}\cdot p\cdot A_{S}}{4\pi R^{2}},$$

where $G_{t}$ and $G_{a}$ denote the gains of the standard horn antenna and power amplifier, respectively, *P* denotes the input power from the signal generator, *R* is defined as the distance from the horn antenna to the RMS, and $A_{s}$ is the effective aperture area of the RMS. The effective receiving aperture $A_{s}$ corresponds to the physical dimensions of the 3 × 3 RMS array. Notably, increasing the size of the RMS array can improve the system’s sensitivity to electromagnetic energy density, as a larger surface area enables the collection of more energy under the same incident power conditions. Additionally, a 100 Ω load is selected.

**Figure 10:**
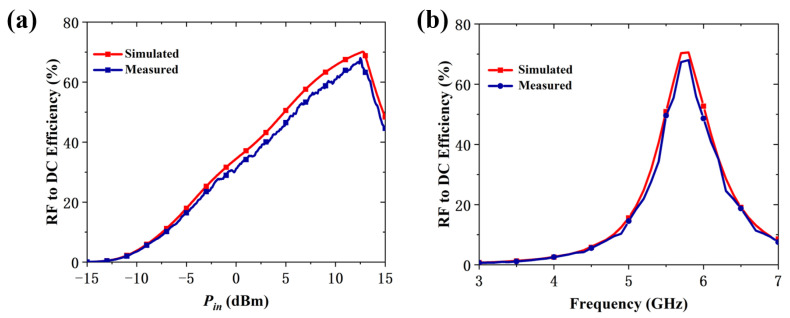
(**a**) Measured RF-to-DC efficiency versus different input power levels at 5.8 GHz. (**b**) Measured RF-to-DC efficiency versus frequency under an input power level of 12.5 dBm.

Figure 10a shows the measured results for RF-to-DC efficiency of the RMS. The efficiency remained above 60% between 9.2 and 13.6 dBm, peaking at 68.3% at an input power of 12.5 dBm. The measured RF-to-DC efficiency of the RMS across various frequencies under an input power of 12.5 dBm is presented in Figure 10b. The efficiency exceeds 50% between 5.5 and 6.0 GHz, reaching a peak of 68.3% at 5.8 GHz. A slight discrepancy is observed between the measured and simulated results. This discrepancy may be attributed to several factors. First, the diode load used in the fabricated prototype may not exactly match the optimal value assumed in simulations, leading to reduced rectification efficiency and overall performance degradation. Second, measurement inaccuracies such as imperfect alignment or environmental noise can introduce deviations. Lastly, fabrication related imperfections, including etching tolerances or substrate warping, may also affect the electromagnetic response of the metasurface, especially in high-frequency applications. Nonetheless, the results confirm that the proposed RMS achieves highly RF-to-DC efficiency over a wider range of input power levels and frequencies.

**Figure 11:**
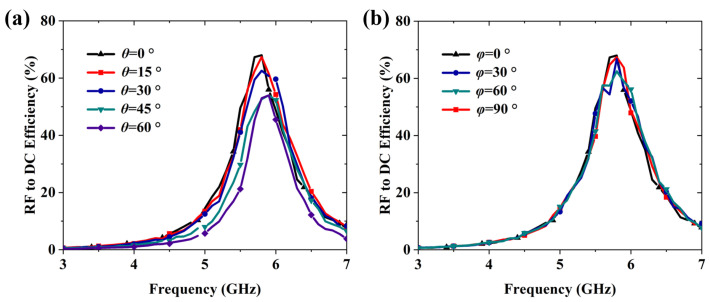
(**a**) Measured RF-to-DC efficiency across frequencies at varying incident angles. (**b**) Measured RF-to-DC efficiency across frequencies under different polarization conditions.

As illustrated in Figure 11a, the proposed RMS maintains over 53.2% RF-to-DC conversion efficiency at 5.8 GHz as the incident angle varies from 0° to 60°. Similarly, Figure 11b demonstrates that the conversion efficiency remains above 62.5% at 5.8 GHz under varying polarization angles from 0° to 90°. These results confirm that the RMS enables wide-angle RF energy harvesting and exhibits robust polarization insensitivity.

Table 1 summarizes the performance comparison between the proposed RMS and existing RF energy harvesting designs reported in the literature. Compared to the previous reports, the proposed RMS maintains high RF-to-DC efficiency by integrating diodes with the metasurface, minimizing energy transmission loss from the lines. This integration also eliminates the need for additional matching networks, simplifying the design process. Moreover, the symmetrical design of the metasurface offers the advantage of polarization insensitivity, enhancing its ability to efficiently collect electromagnetic energy from the environment. In addition to the performance comparison in the table, the proposed RMS also demonstrates effective energy harvesting across a wide incident angle range from 0° to 60°. In conclusion, the proposed RMS demonstrates high RF energy harvesting performance from its surrounding environment.

## 4. Conclusions

In this work, we propose a wide-angle and polarization-insensitive rectifying metasurface designed to harvest RF energy. By adopting the design of placing Schottky diodes on the bottom structure and connecting them to the top structure through four metallized vias, the proposed RMS eliminates the impact of diode soldering on the surface structure and removes the need for a rectifier circuit and matching network. These strategies contribute to enhanced efficiency and structural simplification. Experimental results indicate that the manufactured RMS achieves an RF-to-DC conversion efficiency above 50% within the 5.5–6.0 GHz range, peaking at 68.3% at 5.8 GHz under an input power of 12.5 dBm. Furthermore, the RMS demonstrates stable performance across wide incident angles and polarization states, highlighting its applicability in wireless power transmission and ambient RF energy harvesting scenarios.

## Figures and Tables

**Table 1:** Performance comparison of the proposed design with other related works.

Ref.	Frequency (GHz)	Polarization Insensitive	Optimal Power Level	Need Matching Network	RF to DC Efficiency
[33]	2.4, 5.2, 5.8	No	7 dBm	Yes	46.4% (5.8 GHz)
[35]	5.8	No	7 dBm	Yes	72%
[36]	5.3–6.6	No	5 dBm	Yes	57%
[37]	5.8	Yes	350 mw/${\mathrm{cm}}^{2}$	Yes	55%
[38]	2.4, 5.8	Yes	10 dBm	No	59.0% (5.8 GHz)
This Work	5.8	Yes	12.5 dBm	No	68.3%

## Data Availability

The original contributions presented in the study are included in the article, further inquiries can be directed to the corresponding author.
